# Molecular classification grade 3 endometrial endometrioid carcinoma using a next-generation sequencing–based gene panel

**DOI:** 10.3389/fonc.2022.935694

**Published:** 2022-08-08

**Authors:** Ling Li, Fangfang Chen, Jingcheng Liu, Weifeng Zhu, Liang Lin, Li Chen, Yi Shi, An Lin, Gang Chen

**Affiliations:** ^1^ Department of Gynecological Oncology Surgery, Fujian Medical University Cancer Hospital, Fujian Cancer Hospital, Fuzhou, China; ^2^ Department of Molecular pathology, Fujian Medical University Cancer Hospital, Fujian Cancer Hospital, Fuzhou, China; ^3^ Department of Pathology, Fujian Medical University Cancer Hospital, Fujian Cancer Hospital, Fuzhou, China

**Keywords:** molecular subtype, endometrial carcinoma, POLE ultramutated, microsatellite instability, p53 abnormal

## Abstract

Over the past two decades, the incidence of endometrial cancer (EC) is increasing, and there is a need for molecular biomarkers to predict prognosis and guide treatment. A recent study from The Cancer Genome Atlas suggested to implement the EC analysis by molecular profile for improving diagnosis, prognosis, and therapeutic treatment. In this study, next-generation sequencing was performed on 70 cases of G3 endometrioid ECs (EECs) using an 11-gene panel (TP53, MLH1, MSH2, MSH6, PMS2, EPCAM, PIK3CA, CTNNB1, KRAS, PTEN, and POL) for molecular classification. The molecular classification based on the 11-gene NGS panel identified four molecular subgroups: POLE-ultramutated (n = 20, 28.6%), MSI-H (n = 27, 38.6%), NSMP (n = 13, 18.6%) and TP53mut (n = 10, 14.3%). The NGS method showed 98.6% (69 of 70 cases, kappa value 98%) in concordance with the cases assessed by immunohistochemistry (IHC). Among the seven dead cases, four were MSI-H tumors, two were TP53mut/p53abn tumors, and one was NSMP tumors with an average overall survival (OS) of 14.7 months. TP53mut subgroup showed that poor OS rates and POLE group have favorable prognosis. Our work suggested that the 11-gene panel is suitable for molecular classification in G3 EECs and for guiding prognosis and treatment decisions.

## Introduction

Endometrial cancer (EC) is the sixth most diagnosed cancer in women worldwide (1). The incidence and mortality of EC gradually increases in recent years particularly in industrialized countries. In China, EC is the eighth most diagnosed cancer in women in China with estimated 63.4 thousand newly cases in 2015 (2, 3). The 5-year relative survival rate of EC is 72.8% (4). Bokhman’s dualistic classification broadly classified EC into type I and type II based on histological features and has long been used for clinical diagnostic and therapeutic direction (5). Type I ECs consist mostly of endometrioid ECs (EECs), which are typically low-grade with favorable prognosis. Type II ECs are mostly serous ECs with high-grade and worse prognosis. The 2014 WHO classification for ECs is based on morphologic features, but it has interobserver variation and poor reproducibility especially among high-grade ECs (6, 7). Traditional classification strategies for ECs have proven to be challenging due to the heterogeneous molecular feature of EC. In the last decade, emerging technologies allow the understand of cancer in molecular aspect and bring new diagnostic and therapeutic approaches for cancer into clinical practice (8–10). Like most other types of cancer, EC is a group of heterogeneous tumors with different molecular characteristics. In 2013, The Cancer Genome Atlas (TCGA) Research Network reported four molecular subgroups for EC: POLE (ultramutated), microsatellite instable (MSI, hypermutated), copy number high (CNH), and copy number low (CNL) based on a comprehensive genomic analysis of 373 endometrial carcinomas (11). POLE-ultramutated subgroup characterized by pathogenic POLE exonuclease domain mutations is composed of endometrioid tumors and associated with the most favorable prognosis. Microsatellite instable tumors characterized by deficient MMR (dMMR) with high microsatellite instability (MSI-H) have endometrioid histology and an intermediate prognosis. CNH subgroup characterized by high copy number alterations and *TP53* mutations is associated with poor prognosis and serous histology. CNL subgroup is composed of low-grade endometrioid tumors that are microsatellite-stable and have an intermediate prognosis. The prognostic value TCGA classification has repeatedly been confirmed, offering the possibility of drastically improving the patient management (12–14). However, this classification remains challenging in practice, mainly due to expensive and difficulties of multi-omics analyses. Subsequent classifier, the Proactive Molecular Risk Classifier for Endometrial Cancer (ProMisE), reproduced the four TCGA prognostic subgroups using immunohistochemistry (IHC) for mismatch repair (MMR) proteins and p53 as surrogates of MSI testing and copy number alteration analysis, respectively (15–18).

Given the accumulating evidence on the value of molecular classification on the prognosis prediction and personized treatment for EC, molecular classification is included in the fifth edition of the WHO classification of tumors of the female genital tract, the National Comprehensive Cancer Network (NCCN) and the ESGO/ESTRO/ESP guidelines for EC (19–21). Next-generation sequencing (NGS) technology is a promising method enabling large-scale genomic sequencing (22), which may have advantageous in accuracy and time and cost efficiency for molecular classification of EC. However, few studies have reported the utility of NGS panel alone for EC molecular classification. In this study, we applied NGS technology to determine the molecular subtypes of G3 EECs using a 11-gene panel and compared with IHC approaches.

## Materials and methods

### Case selection and histologic review

Our retrospective cohort consists of 70 patients with grade 3 EEC treated at Fujian Provincial Cancer Hospital from June 2018 to May 2021. According to the revised standards of the 5th edition of the WHO classification in 2017, all hematoxylin and eosin slides were reviewed by two pathologists (GC and JL) and the diagnosis of grade 3 EEC was confirmed on the basis of morphologic features. Clinical and pathology database of patients were collected, including age, tumor size, tumor size, FIGO stage, lymph node status, and LVSI. This retrospective study was approved by the Ethics Committee of Fujian Provincial Cancer Hospital.

### Next-generation sequencing

For NGS assay, six to eight formaldehyde-fixed and paraffin embedded tissue (FFPE) sections of 5- to 10-µm-thin size containing more than 50% tumor cells were used, and total DNA was extract from FFPE using a TIANamp FFPE DNA Kit (TianGen, Beijing). DNA concentration was measured using a Quantus Fluorometer (Promega, Shanghai, China). Library was prepared using a RingCap amplicon library kit for the custom-designed 11-gene panel (SpaceGen, Xiamen, China). The panel targets the whole coding regions of *TP53*, *MLH1*, *MSH2*, *MSH6*, *PMS2*, and *EPCAM* and hotspot region of *PIK3CA*, *CTNNB1*, *KRAS*, *PTEN*, and *POLE* exonuclease domain. MSI status was determined by the NGS method including five mononucleotide repeat MSI markers (BAT25, BAT26, NR-21, NR-24, and MONO-27) within the 11-gene panel. Sequencing was performed on a Miseq Dx platform (Illumina, USA). Raw sequencing data were trimmed and aligned to human hg19 reference genome using Trimmomatic (version 0.36) and Burrow-Wheeler Aligner (BWA) (version 0.7.17). Next, aligned reads were processed to call SNVs and small Indels using Pisces (version 5.2.9), followed by variants annotation using ANNOVAR (version 20180426). MSIsensor-pro package (version 1.2.0) was used to identify MS-stable (MSS, no MSI makers), MSI-low (MSI-L, one MSI maker), and MSI-high (MSI-H, two or more MSI makers) tumors without matched normal sample (20).

### Immunohistochemistry

IHC was performed on 3-µm-thick FFPE sections using primary antibodies as follows: MLH1 (clone: MAB-0838, 1:300, Maxim, Fuzhou, China), PMS2 (clone: MAB-0859, 1:300, Maxim, Fuzhou, China), MSH2 (clone: MAB-0836, 1:300, Maxim, Fuzhou, China), MSH6 (clone: MAB-0831, 1:300, Maxim, Fuzhou, China), and p53 (clone: MAB-0674, 1:300, Maxim, Fuzhou, China). Tumors showed any loss of nuclear expression of the four MMR proteins (MLH1, PMS2, MSH2, and MSH6) were designated as MMR-deficient (MMRd). P53 expression and mutant patterns were interpreted as abnormal/aberrant/mutation-type (p53abn) or wild type (p53wt) following previous published criteria (19). Three patterns were considered aberrant: (1) strong diffuse nuclear staining in > 75% of tumor cells; (2) complete absence of staining; and (3) cytoplasmic staining. All the IHC images for MMR status and p53 expression were reviewed independently by two pathologists (GC and JL).

### MLH1 promoter methylation testing

Genomic DNA from 42 FFPE tumor samples was extracted by a TIANamp FFPE DNA Kit (TianGen, Beijing), and Bisulphite treatment was performed on 1 µg of DNA with the EpiTect Bisulfite kit (Qiagen, Valencia, CA, United States). Bisulfite-converted DNA was amplified for MLH1 (a methylation-independent reaction for normalization), and methylation status was analyzed on the basis of previously reported method (23).

### Statistical analysis

Statistical analysis was performed in SPSS 22.0. Associations of clinicopathological parameters with molecular subtypes were compared using chi-squared test in. For the concordance of NGS-based and IHC-based methods, kappa value was calculated.

## Results

### Clinicopathological characteristics

Seventy patients with high-grade EEC were 29–67 years old at onset, with a median age of 54.5 years. All patients were operated by “hysterectomy, double ovaries and fallopian tubes and pelvic lymph node dissection”. Postoperatively, tumor diameter of 0.5–9 cm was seen (except for two cases which showed rough mucosa and no obvious tumor mass); as for the site of onset, 13 cases were in the lower part of the uterine body and 57 cases were in other parts of the uterine body. In our group, 33 cases had tumor infiltration depth of more than one-half of muscle layer, 28 cases had cancer thrombus in the vasculature, and 20 patients had lymph node metastasis. According to the 2009 International FIGO staging criteria, there are 29 patients in stage I, 14 patients in stage II, 24 patients in stage III, and three patients in stage IV. In terms of postoperative adjuvant therapy, 41 cases were treated with radiotherapy, 18 with adjuvant chemotherapy, three with adjuvant local radiotherapy, and eight without adjuvant therapy; as for concomitant diseases, eight patients had a history of hypertension (grades 2–3) and two patients had diabetes mellitus; regarding the family history of malignancy, there were six cases of patients with cancer in their immediate family. The baseline clinicopathological characteristics of the 70 G3 EEC patients are shown in **Table 1**. The univariable associations of their molecular subtypes with clinicopathological parameters were calculated. All clinicopathological parameters (age, tumor size, myometrial invasion, myometrial invasion, FIGO stage, lymph node status, and LVSI) are shown no significant relationship with molecular subtypes (*P* > 0.05).

**Table 1:** Univariable associations of molecular subtypes with clinicopathological parameters.

	Total	POLE	MSI-H	NSMP	TP53mut	P-value
**Number of Patients**	70	20 (28.57%)	26 (37.14%)	14 (20%)	10 (14.29%)	
**Age, years**						0.533
<60	60 (85.71%)	17 (85%)	21 (80.77%)	12 (85.71%)	10 (100%)	
≥60	10 (14.29%)	3 (15%)	5 (19.23%)	2 (14.29%)	0 (0%)	
**Tumor size, cm**						0.511
≤2	10 (14.29%)	1 (5%)	3 (11.54%)	4 (28.57%)	2 (20%)	
2–5	43 (61.43%)	15 (75%)	15 (57.69%)	7 (50%)	6 (60%)	
≥5	17 (24.29%)	4 (20%)	8 (30.77%)	3 (21.43%)	2 (20%)	
**Myometrial invasion**						0.063
<50%	37 (52.86%)	9 (45%)	11 (42.31%)	8 (57.14%)	9 (90%)	
≥50%	33 (47.14%)	11 (55%)	15 (57.69%)	6 (42.86%)	1 (10%)	
**FIGO stage**						0.727
I/II	43 (61.43%)	14 (70%)	14 (53.85%)	9 (64.29%)	6 (60%)	
III/IV	27 (38.57%)	6 (30%)	12 (46.15%)	5 (35.71%)	4 (40%)	
**Lymph node status**						0.173
Negative	50 (71.43%)	18 (90%)	16 (61.54%)	9 (64.29%)	7 (70%)	
Positive	20 (28.57%)	2 (10%)	10 (38.46%)	5 (35.71%)	3 (30%)	
**LVSI**						0.520
Negative	42 (60%)	12 (60%)	13 (50%)	10 (71.43%)	7 (70%)	
Positive	28 (40%)	8 (40%)	13 (50%)	4 (28.57%)	3 (30%)	

### Molecular typing based on NGS detection

**Figure 1:**
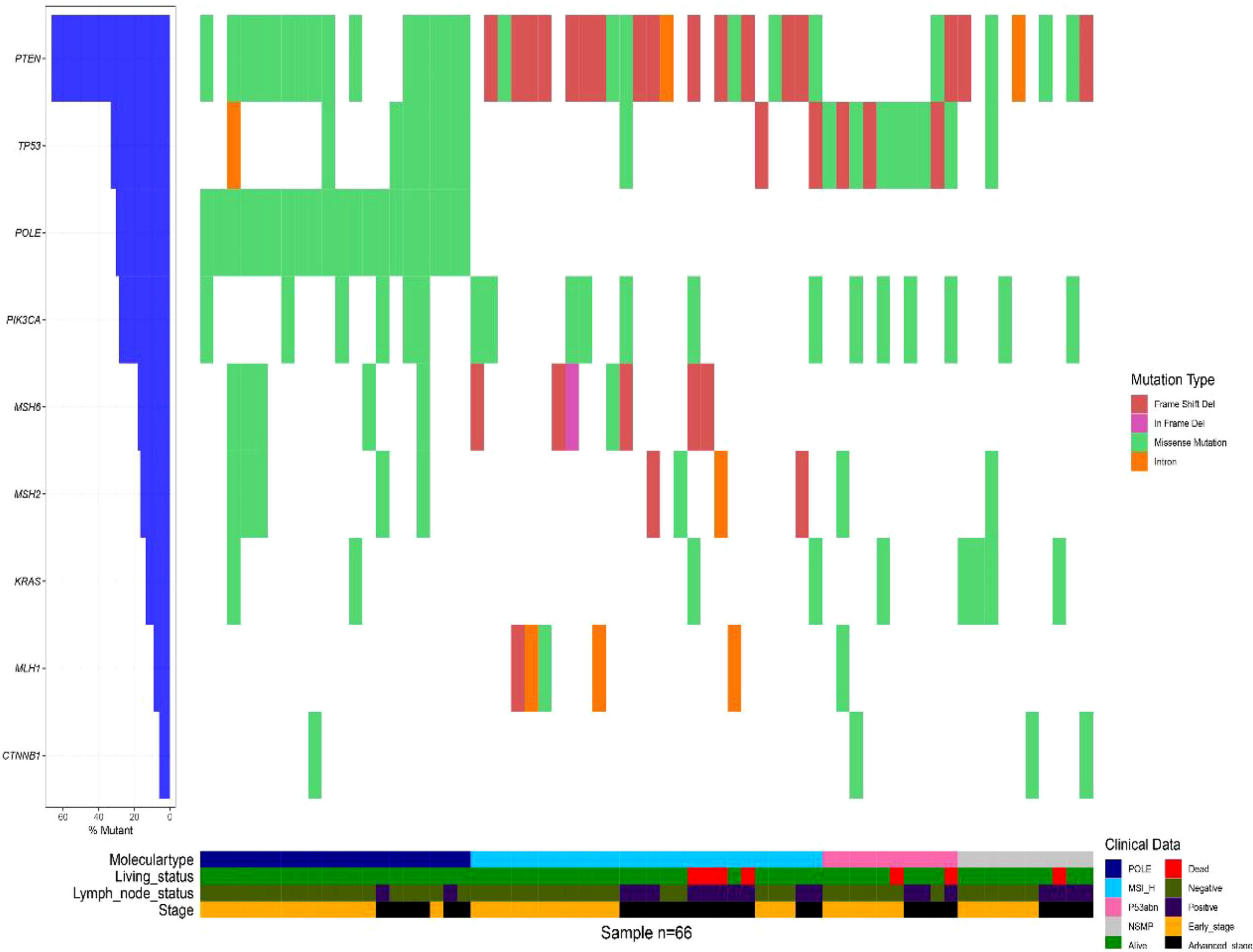
Gene mutational profile 70 G3 EECs.

The molecular classification using NGS panel for 70 G3 EECs was shown in **Table 1**. The molecular classification based on the 11-gene NGS panel identified four molecular subgroups: POLE-ultramutated (n = 20, 28.6%), MSI-H (n = 27, 38.6%), NSMP (n = 13, 18.6%), and TP53mut (n = 10, 14.3%). On the basis of the 11-gene NGS panel, pathogenic mutations of *PTEN*, *TP53*, *PIK3CA*, *CTNNB1*, and *KRAS* were found in 54.3%, 28.6%, 22.9%, 5.7%, and 12.9% of the total cases, respectively, as seen in **Table 2** and **Figure 1**.

**Table 2:** Molecular classification of 70 G3 EECs.

		11-gene NGS panel				TotalN (%)
		POLE	MSI-H	NSMP	TP53mut	
IHC	POLE	20	0	0	0	20 (28.6)
	MMRd	0	26	0	0	26 (37.1)
	NSMP	0	1	13	0	14 (20)
	p53abn	0	0	0	10	10/14.3
Total, N (%)		20 (28.6)	27 (38.6)	13 (18.6)	10 (14.3)	70 (100)
PTEN*mut*, N (%)		14 (60)	17 (63)	5 (38.5)	2 (20)	38 (54.3)
TP53*mut*, N (%)		7 (35)	3 (11.1)	0 (0)	10 (100)	20 (28.6)
PIK3CA*mut*, N (%)		6 (30)	4 (14.8)	2 (15.4)	4 (40)	16 (22.9)
CTNNB1*mut*, N (%)		1 (5)	0 (0)	2 (15.4)	1 (10)	4 (5.7)
KRAS*mut*, N (%)		2 (10)	3 (11.5)	3 (23.1)	1 (10)	9 (12.9)

MSI-H, high microsatellite instability; MMRd, MMR proteins deficient; NSMP, no specific molecular profile; TP53mut, TP53 pathogenic mutation; p53abn, p53 protein abnormal; PTENmut, PTEN pathogenic mutation; PIK3CAmut, PIK3CA pathogenic mutation; CTNNB1mut, CTNNB1 pathogenic mutation; KRASmut, KRAS pathogenic mutation.

Among the 20 cases in the POLE-ultramutated subgroup, the most common hotspot mutations (P286R and V411L) were found in 10 (50%) and seven (35%) tumor samples, respectively (**Table 2**). In addition, A456P, P436R, and Y473C were each present in one case (**Table 2**). Furthermore, the most commonly hotspot mutation (P286R and V411L) covers 85% of the POLE-ultramutated cases. In addition, high mutational load and multiple molecular features were observed in the POLE-ultramutated subgroup with on two MMRd/MSH-H cases and seven TP53mut/p53abn cases (**Table 3** and **Figure 1**).

**Table 3:** Molecular characteristics of POLE-ultramutated G3 EECs.

	n (%)
POLE pathogenic mutations	
p.P286R	10 (50)
p.V411L	7 (35)
p.Y473C and P286R	1 (10)
p.A456P or p.P436R	2 (10)
MSI-H by NGS	2 (10)
MMR pathogenic mutations by NGS	7 (35)
MMRd by IHC	1 (5)
TP53 pathogenic mutations by NGS	7 (35)
p53abn by IHC	6 (30)

MSI-H, high microsatellite instability; MMRd, MMR proteins deficient; p53abn, p53 abnormal.

### MSI-NGS testing

**Figure 2:**
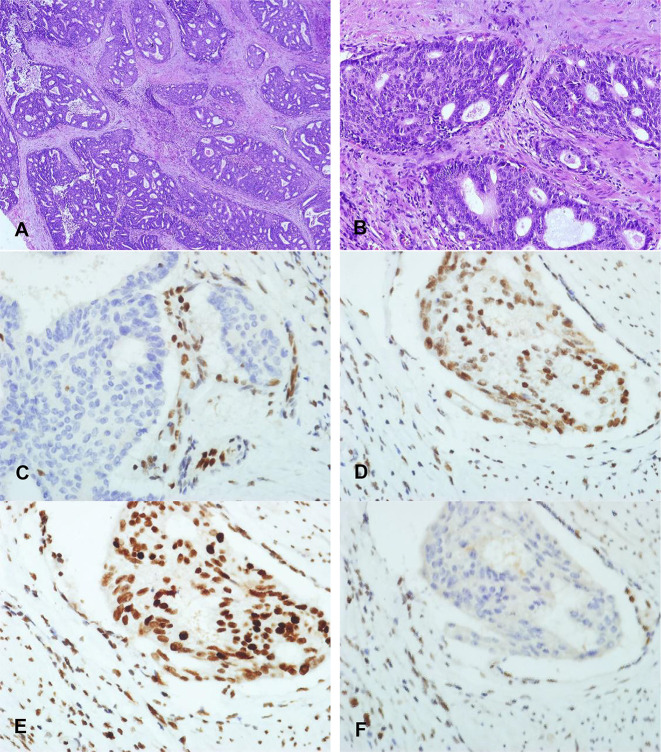
Immunohistochemical staining characteristics of EECs of MLH1 methylation. **(A)** X40 and **(B)** X100: Morphology of high-grade endometrioid adenocarcinoma with MLH1 methylation. **(B)** MLH1 protein expression pattern is completely absent of high-grade endometrioid adenocarcinoma with MLH1 methylation (X200). **(C–F)** MSH2, MSH6, and PMS2 protein are positive expression pattern of high-grade endometrioid adenocarcinoma with MLH1 methylation (X200).

We identified 28 MSI-H (40%), four MSI-L (5.7%), and 38 MSS (54.3%) EECs, respectively. Twenty-seven of the 28 MSI-H tumors were classified as MSI-H subtype with only one tumor that belongs to POLE subtype. Among these MSI-H tumors, two cases showed normal MMR staining by IHC and were classified to the NSMP group, indicating the strong consistency between the MSI-NGS and MMR-IHC methods. Further MLH1 methylation testing showed that one of the two doubtable cases was high-methylated (**Figure 2**) and the other was not methylated. The not methylated MSI-H case had two instable repeat loci (BAT-26 and MONO-27) with an additional MSH2 nonsense mutation (p.Q344X, mutation allele frequency: 48.17%) tested by NGS but showed normal MMR staining by IHC.

There were twenty (28.6%) tumors harboring somatic pathogenic mutations of *TP53* identified by NGS, of which, 10 were classified to TP53mut, eight were POLE-ultramutated, and two were MSI-H (**Table 4**). Among these cases, five tumors showed normal p53 staining by IHC (three cases in the POLE-ultramutated group and two cases in the MSI-H group, see **Table 4**). In addition, there was one sample identified TP53wt by NGS but showed p53 abnormal staining by IHC.

**Table 4:** Comparison of NGS and IHC for TP53 (p53) status assessment.

	TP53wt by NGS(n = 50)	TP53mut by NGS (n = 20)			Total
		POLE-ultramutated	MSI-H	TP53mut	
p53wt by IHC	49	3	2	0	
p53abn by IHC	1	5	0	10	
Total	50	8	2	10	70

TP53mut, TP53 pathogenic mutation; TP53wt, TP53 wild type; p53wt, p53 protein present; p53abn, p53 protein abnormal.

### IHC

**Figure 3:**
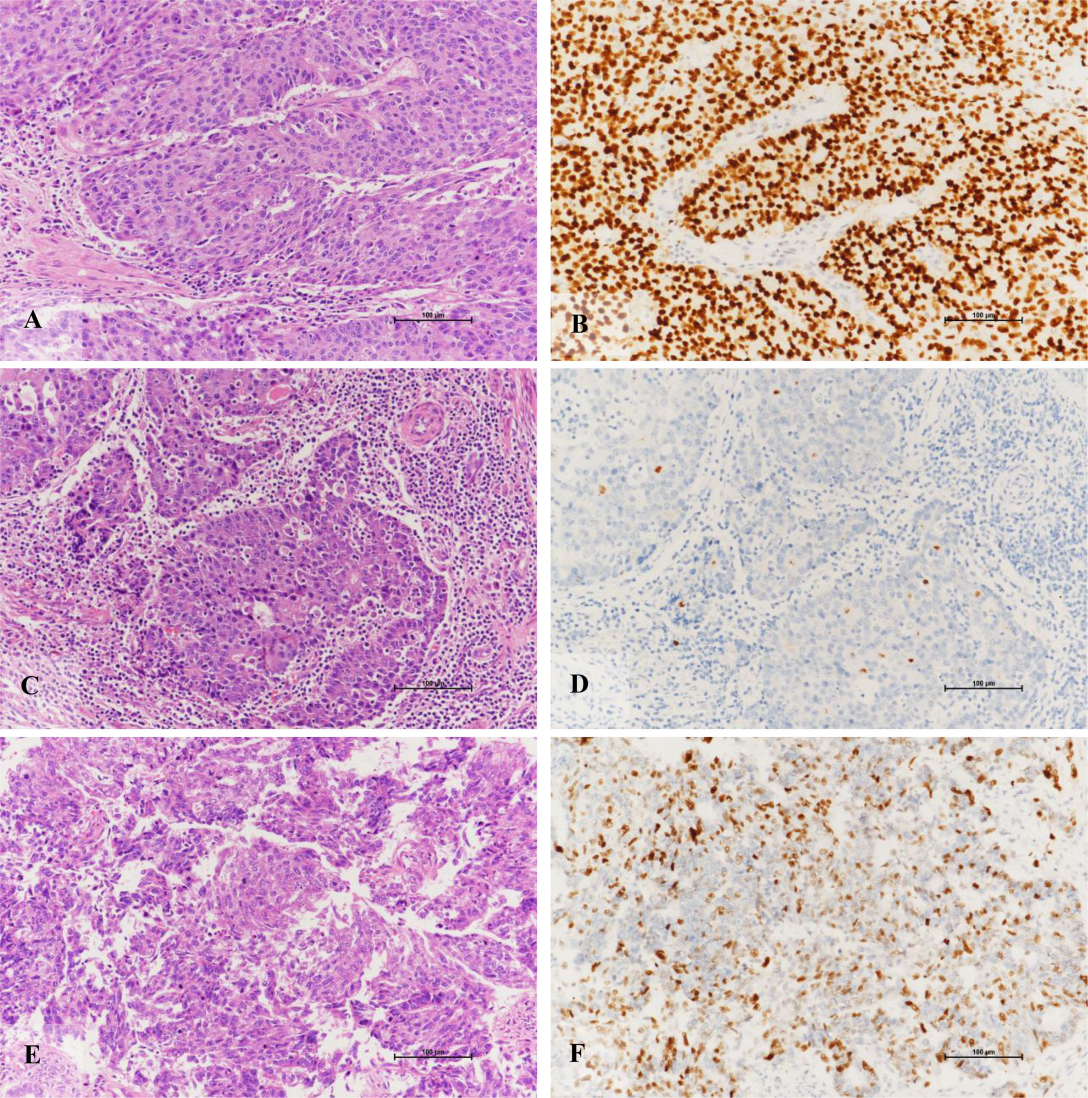
Immunohistochemical staining characteristics of EECs of TP53 subtypes. **(A, B)** Morphology of high-grade endometrioid adenocarcinoma with TP53 gene mutation and its diffuse positive expression pattern of p53 protein (X200). **(C, D)** High-grade endometrioid adenocarcinoma with TP53 gene mutation is rich in lymphocyte stroma and its p53 protein expression pattern is completely absent (X200). **(E, F)** The morphology of high-grade endometrioid adenocarcinoma without mutation of TP53 gene and its positive expression pattern of p53 protein (X200).

The molecular classification based on POLE sequencing and MMR and p53 IHC identified four parallel subgroups: POLE-ultramutated (n = 20, 28.6%), MMRd (n = 26, 37.1%), NSMP (n = 14, 20%), and p53mut (n = 10, 14.3%) (**Figure 3**). The molecular classification using NGS panel and IHC for 70 G3 EECs was shown in **Table 1**. The NGS method showed 98.6% (69 of 70 cases) in line with the IHC method (kappa value 98%).

### Morphological characteristics based on molecular typing

**Figure 4:**
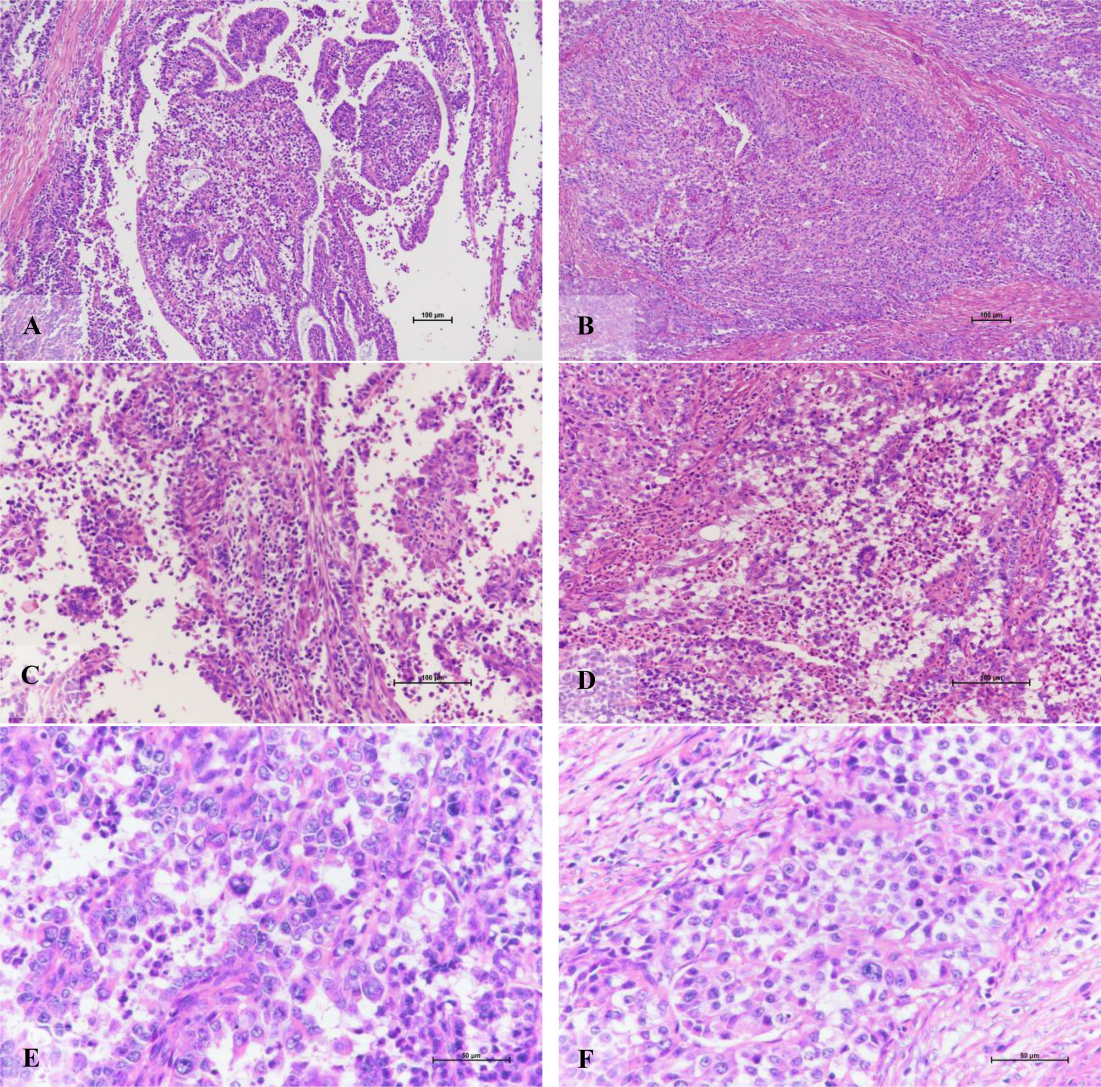
Morphological characteristics of POLE-EDM endometrioid adenocarcinoma. **(A)** The tumor cells grow in glandular tube and papillary shape (X100). **(B)** The tumor cells present a solid, nest-like muscular layer infiltration pattern (X100). **(C)** The mesenchyme adjacent to the tumor cell nest, rich in tumor-associated lymphocyte areas (X200). **(D)** Excluding necrotic area, tumor stroma is rich in tumor-associated neutrophil area (X200). **(E, F)** tumor giant cells of varying sizes and rare pathological mitotic figures (X400).

Morphologically, the POLE-EDM subtype is similar to the morphological characteristics of serous carcinoma, but there are still subtle differences. It is mainly manifested in the areas of tumors that are easy to see sheet necrosis, and there are different numbers of bizarre polynucleoma giant cells and tumors. Tumor-related lymphocytes and neutrophils can be seen in the necrotic area except for the tumorous stroma (**Figure 4**). Partially visible deep muscle infiltration (11 of 20, 55.0%), intravascular tumor thrombus (8 of 20, 40.0%), and lymph node metastasis (2 of 20, 10.0%).

**Figure 5:**
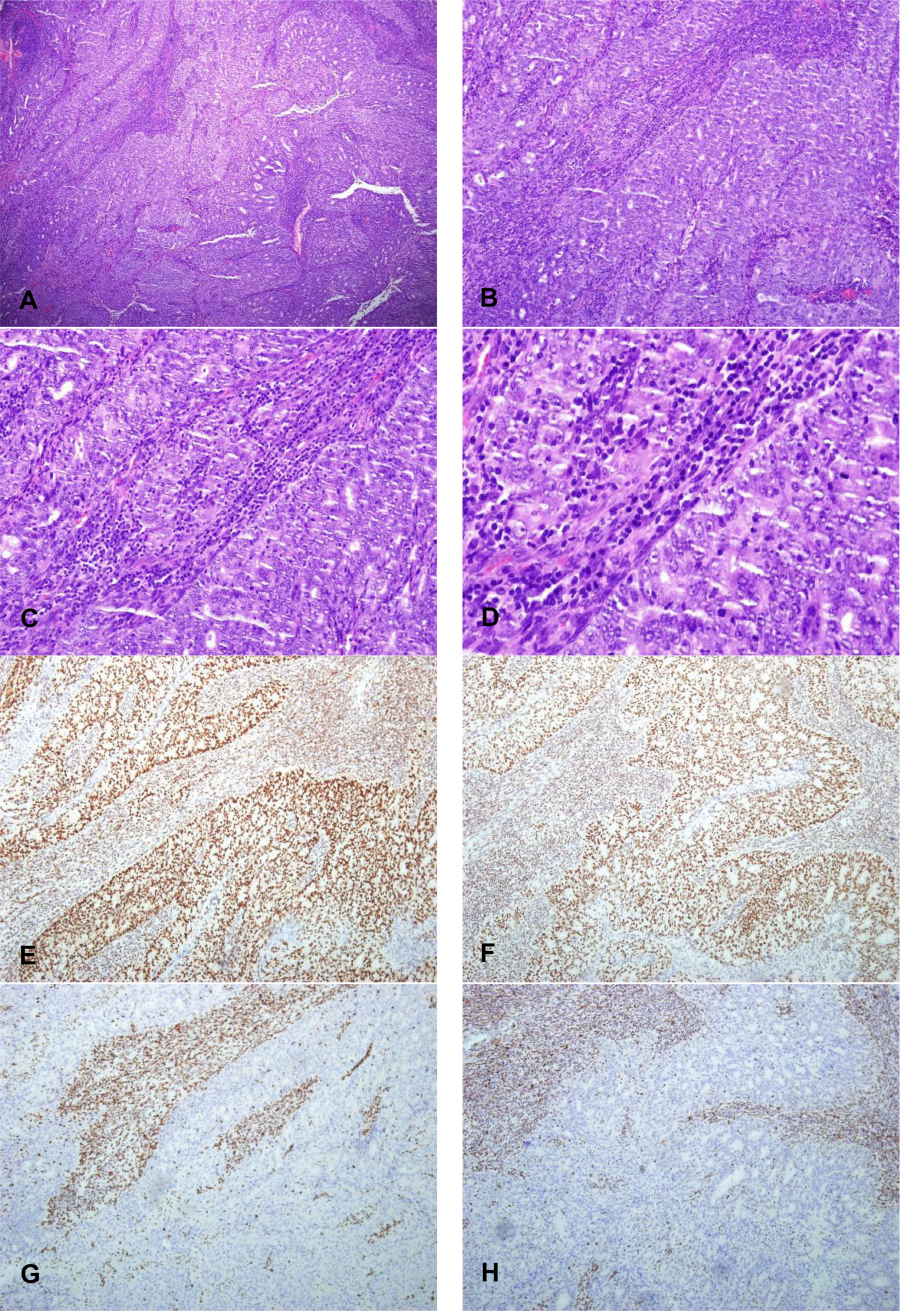
The morphology of high-grade endometrioid adenocarcinoma of MSI-H subtypes and its expression pattern of MMR protein (X100). **(A)** X40 and **(B)** X100: The morphological characteristics of MSI-H subtype are similar to high-grade serous carcinoma. Sheet necrosis is easy to see; the stroma is rich in inflammatory cells dominated by lymphocytes **(C)** X200 and **(D)** X400: The nucleus is significantly atypia, and the mitotic image is easy to see of EECs of MSI-H subtypes. **(E, F)** MLH1 and PMS2 proteins expression pattern are positive expression pattern (X100). **(G, H)** MSH2 and MSH6 proteins expression pattern are completely absent (X100).

The morphological characteristics of MSI-H subtype are similar to high-grade serous carcinoma. Sheet necrosis is easy to see, the stroma is rich in inflammatory cells dominated by lymphocytes, the nucleus is significantly atypia, and the mitotic image is easy to see (**Figure 5**). Most tumors have infiltrated more than one-half of the muscle layer (15 of 26, 57.7%), and some have vascular invasion (13 of 26, 50%), but lymph node metastasis is less (10 of 26, 38.5%).

**Figure 6:**
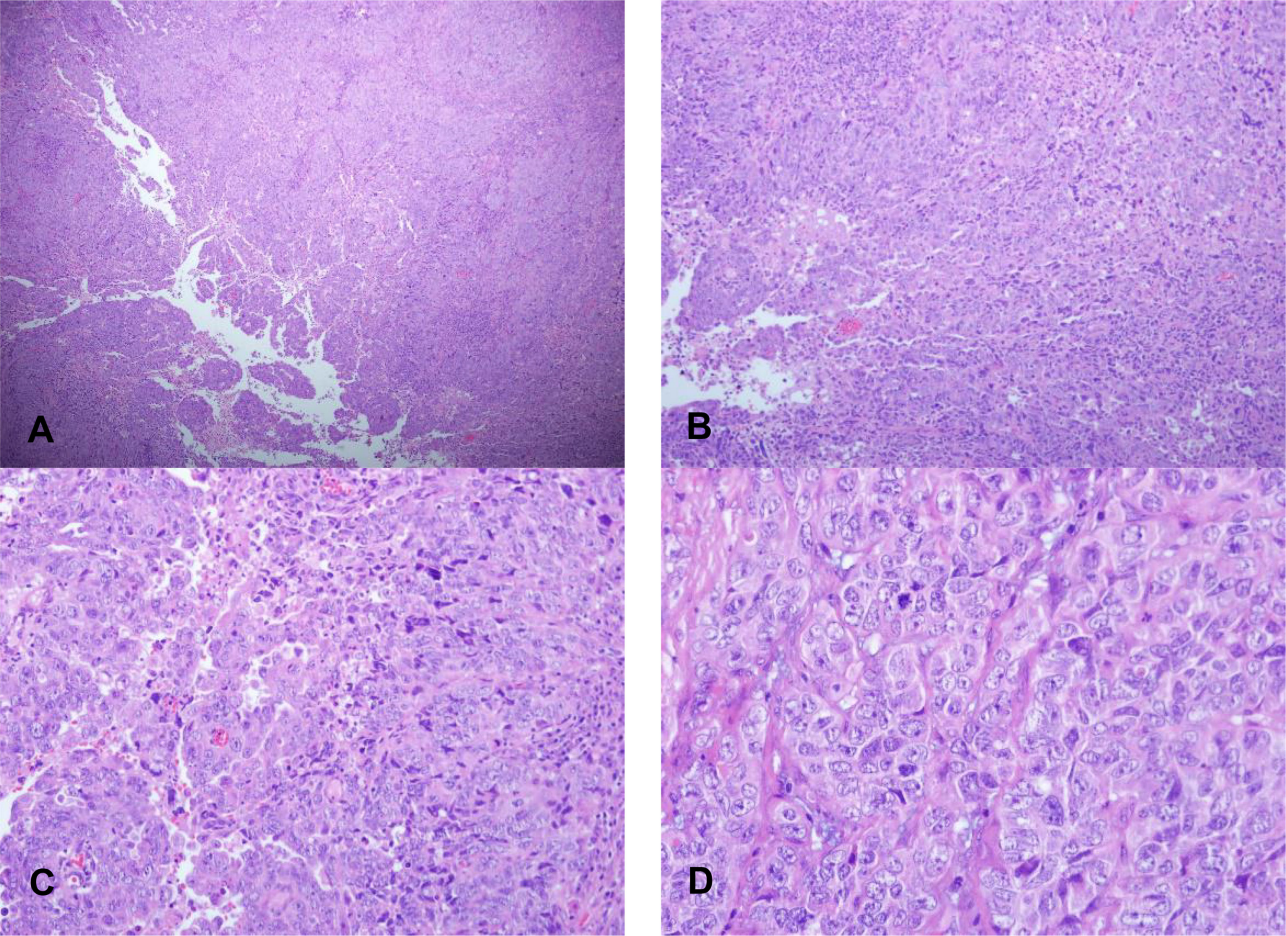
Morphological characteristics of EECs of TP53 subtypes. **(A)** X40 and **(B)** X100: The coexistence of solid and adenoid tumors of EECs of TP53 subtypes. **(C)** X200 and **(D)** X400: The contrast between nuclear atypia and tissue structure is slightly lower with mitotic images.

The TP53 mutant is easy to see the coexistence of solid and adenoid tumors. Compared with serous carcinoma, the contrast between nuclear atypia and tissue structure is slightly lower, and mitotic images are easy to see (**Figure 6**). In addition, there was one case with tumor infiltration depth exceeding one-half of the muscle layer, three cases with vascular invasion, three cases with lymph node metastasis, and one case with positive cytology of peritoneal washing fluid.

## Discussion

Molecular classification strategies (multi-omics) for endometrial carcinoma described in the initial TCGA study are expensive, time-consuming, and technically challenging for clinical use. Subsequent large cohort studies (ProMisE and PORTEC) developed clinically feasible molecular classifiers based on surrogate strategies including POLE sequencing, MMR-IHC, p53-IHC, or TP53 sequencing. NGS is a promising method enabling large-scale genomic sequencing and advantageous in accuracy and time and cost efficiency for molecular classification of EC. In this study, we designed an NGS panel and aimed to evaluate its potential clinical utility as an EC molecular classification tool. Our study population consists of 70 G3 EECs, mostly (85.7%) aged <60 years old. We identified four molecular subgroups: POLE-ultramutated (28.6%), MSI-H (38.6%), NSMP (18.6%), and TP53mut (14.3%) using the 11-gene NGS panel, indicating the high heterogeneity in molecular subtypes of G3 EECs, which has been reported in several recent studies. In the retrospective cohort of Bosse’s study on 381 G3 EECs, there were 49 (12.9%) POLE, 79 (20.7%) p53abn, 115 (30.2%) NSMP, and 138 (36.2%) MMRd tumors. The higher percentage of POLE-ultramutated cases and lower percentage of p53abn cases in our study may be on account of younger age at diagnosis of the patients (24, 25). As reported in the 257 young (<50 years old) women with EC from the ProMisE cohort, more POLE-mutated cases and less in p53abn cases were observed compared with other non-age-stratified cohorts (26). Furthermore, the NGS method showed 98.6% (69 of 70 cases) in line with the IHC method (kappa value 98%). Similar results have been reported by Huvila et al. They compared the results of NGS-based (Foundation One CDx with 114 repeat loci) and IHC-based (ProMisE) molecular classification for ECs. The result showed excellent agreement in 98.1% of cases between MSI-NGS and MMR-IHC analyses with one tumor described MSS by NGS but loss of MLH1 and PMS2 expression by IHC (27). In addition, consistent with previous findings, the most commonly hotspot mutation (P286R and V411L) covers 85% of the POLE-ultramutated cases.

Recently, with the emergence of molecular classification and approval of several immune checkpoint inhibitors for EC, MSI has become a part of the standard molecular testing for EC. In tumor cells, aberrant expression of MMR proteins causes microsatellite instability that can be assessed by the IHC, PCR, and NGS methods. In the TCGA study, MSI status was determined using PCR amplification and capillary electrophoresis on seven repeat loci and used to define the MSI-H (hypermutated) subgroup (11). MMR-IHC, a surrogate method for MSI testing, showed a high diagnostic accuracy in recent large cohort studies (26). As a promising strategy for MSI testing, NGS method enables detection of hundreds of MSI markers and MMR gene mutations. A recent study has reported that using NGS for the detection of MMR gene mutations to identify MSI hypermutated ECs is insufficient for MMR gene mutation that is not the only factor leading to MSI-H/MMRd (28). The NGS panel used in this study was designed to detect somatic mutations of the five MMR genes and instability of five microsatellite repeat loci using multiplex amplicon sequencing. We identified 28 MSI-H ECs, 27 of which were classified as the MSI-H subgroup. Among these, two cases showed normal MMR expression and were classified to the NSMP group by IHC, indicating the strong consistency (26 of 28, 92.8%) between the MSI-NGS and MMR-IHC methods. Similar results have been reported by Huvila et al (27). They compared the results of NGS-based (Foundation One CDx with 114 repeat loci) and IHC-based (ProMisE) molecular classification for ECs. The result showed excellent agreement in 98.1% of cases between MSI-NGS and MMR-IHC analyses with one tumor described MSS by NGS but loss of MLH1 and PMS2 expression by IHC (27). We further confirmed that one of the two doubtable ECs was MLH1 promoter hypermethylated tumor. The non-methylated MSI-H case had two instable repeat loci (BAT- 26 and MONO-27) and an additional MSH2 p.Q344X mutation with an allele frequency of 48.17% tested by NGS; further analysis is needed to identify whether it is a Lynch syndrome tumor. These results indicated NGS panel is a useful diagnostic tool for MSI-H ECs and may be more accuracy than IHC due to interobserver variability (29). In addition, when doubtable results exist, MLH1 promoter methylation analysis is essential for validation.

There were 20 (28.6%) tumors harboring somatic pathogenic mutations of TP53 identified by NGS, of which 10 were classified to TP53mut, eight were POLE-ultramutated and two were MSI-H. Among these cases, five tumors showed normal p53 staining by IHC, which were referred to as “multiple-classifier”. In addition, there was one sample of the POLE subgroup identified TP53wt by NGS but showed p53 abnormal staining by IHC. Consistently, all cases in the TP53mut group were observed abnormal expression of p53 protein by IHC. Interestingly, the six inconsistent cases with TP53 mutations or p53 abnormal staining were “multiple-classifier” ECs (four cases in the POLE-ultramutated group and two cases in the MSI-H group). This is likely because that more TP53 mutations could be identified by NGS compared with p53 IHC (30). TP53 mutations or abnormal expression in the “multiple-classifier” cases may occur secondary to POLE mutation of MMRd during tumor progression that is confirmed in previous findings (31). Furthermore, TP53 mutations may not impact the expression of p53 protein (32). A recent study reported that the concordance between p53 IHC and TP53 mutation was 155 of 168 (92.3%) overall and 117 of 123 (95.1%) after excluding MMRd and POLEmut EC, suggesting a high proportion of inconsistent cases in “multiple-classifier” ECs 30. Future studies are needed to better understand the inconsistency between TP53 sequencing and p53 IHC in “multiple-classifier” ECs.

In summary, in our study, the designed 11-gene NGS panel showed excellent availability for EC molecular classification as compared with IHC approaches. The NGS panel combined mutation and MSI analyses provide an efficient and accurate molecular classifier for EC. However, there are a few limitations of this study. For example, the cohort used in this study is not particularly large, which might affect the robust of statistics used in this study. In addition, although the molecular classification is important, it might be also important to study the drugs specific for subtypes using some drug repositioning or other methods (33, 34). Moreover, this study is mostly at DNA level, the integration of multi-omics data might provide more useful subtyping. Finally, it might be better to introduce single-cell technologies (35), because EC is a quite heterogeneous cancer. In the future, more work should be done based on larger cohorts to validate its prognostic value before clinical application.

## Data availability statement

The original contributions presented in the study are included in the article/supplementary material Further inquiries can be directed to the corresponding author.

## Ethics statement

The studies involving human participants were reviewed and approved by the Ethics Committee of Fujian Provincial Cancer Hospital. Written informed consent for participation was not required for this study in accordance with the national legislation and the institutional requirements.

## Author contributions

All authors contributed to the study conception and design. GC conceived the project. LL implemented the experiments and analyzed the data. FC, JCL, WFZ, LLin, LC, AL, and YS prepared the data and performed literature search. LLi, FC, and GC wrote the manuscript. All authors approved the final manuscript. LLi and FC contributed equally to this work. All authors contributed to the article and approved the submitted version.

## Funding

This work was supported by Fujian Provincial Science and Technology Department Planning Project, Grant Number 2019L3018.

## Acknowledgments

We greatly appreciate the assistance on the statistical analysis of the study from Yan Chen (Xiamen Spacegen Co., Ltd.).

## Conflict of interest

The authors declare that the research was conducted in the absence of any commercial or financial relationships that could be construed as a potential conflict of interest.

## Publisher’s note

All claims expressed in this article are solely those of the authors and do not necessarily represent those of their affiliated organizations, or those of the publisher, the editors and the reviewers. Any product that may be evaluated in this article, or claim that may be made by its manufacturer, is not guaranteed or endorsed by the publisher.
